# Prognosis and survival analysis of patients with pancreatic cancer: retrospective experience of a single institution

**DOI:** 10.1186/s12957-021-02478-x

**Published:** 2022-01-07

**Authors:** Qi Li, Zijian Feng, Ruyi Miao, Xun Liu, Chenxi Liu, Zhen Liu

**Affiliations:** Department of General Surgery, Shengjing Hospital of China Medical University, 110004, No. 36 Sanhao Street, Heping District, Shenyang, Liaoning Province China

**Keywords:** Pancreatic cancer, Clinicopathological factor, Therapy method, Prognosis, Cox regression analysis

## Abstract

**Background:**

The overall survival of patients  with pancreatic cancer is extremely low. Despite multiple large-scale studies, identification of predictors of patient survival remains challenging. This study aimed to investigate the prognostic factors for pancreatic cancer.

**Methods:**

The clinical data of 625 patients with pancreatic cancer treated at Shengjing Hospital of China Medical University from January 2013 to December 2017 were collected.

**Results:**

Of 625 patients, 569 were followed from 1 to 75 months. The median overall survival was 9.3 months. The overall 1-, 3-, and 5-year survival rates were 37.8%, 15.1%, and 10.5%, respectively. Cox proportional hazards model indicated that baseline carbohydrate antigen 199 level, neutrophil-lymphocyte ratio, operative procedure, lymph node metastasis, number of distant organ metastasis, and postoperative adjuvant chemotherapy were independent prognostic factors of patients with pancreatic cancer. Baseline carbohydrate antigen 199 level, degree of weight loss, operative procedure, lymph node metastasis, number of distant organ metastasis, and postoperative adjuvant chemotherapy were independent prognostic factors of pancreatic head cancer subgroup. Baseline carbohydrate antigen 199 level, carcinoembryonic antigen level, total bilirubin level, neutrophil-lymphocyte ratio, peripancreatic invasion, number of distant organ metastasis, and postoperative adjuvant chemotherapy were independent prognostic factors of the pancreatic body/tail cancer subgroup.

**Conclusions:**

Higher carbohydrate antigen 199 levels, neutrophil-lymphocyte ratio, lymph node metastasis and distant organ metastasis predict a poor prognosis in patients with pancreatic cancer. Early detection, early radical surgery and adjuvant chemotherapy are needed to improve prognosis for this deadly disease.

## Background

Pancreatic cancer (PC) is one of the most lethal malignant tumors of the digestive system. Its characteristics include hidden symptoms, rapid progression, difficult early diagnosis, short survival time, and poor prognosis. The incidence of PC increases annually. In 2014, a study [1] reported that there were 52,000 new male patients with PC and 40,000 female patients with PC in China. The mortality rate of PC in China ranked seventh among all cancer-related deaths in men and eighth in women. Another study reported that PC will become the second leading cause of cancer-related deaths by 2030 in the United States [2].

Unfortunately, population-based studies in several parts of the world have shown limited survival improvement in patients with nonmetastatic PC over the years [3]. The situation is even worse in the advanced stages of the disease [4]. Most patients are already in the advanced stage of the disease when they are diagnosed, and only 15–20% of the tumors are resectable [5]. Study [6] have shown that, despite radical resection of pancreatic adenocarcinoma, the 5-year survival rate is only 10–25%. More than 80% of patients diagnosed with PC are not suitable for surgical treatment owing to local or distant metastasis [7, 8]. In most patients with advanced disease, effective treatment with FOLFIRINOX or nab-paclitaxel plus gemcitabine chemotherapy provides only limited survival benefit [9, 10]. Thus, the diagnosis and treatment of PC is challenging, and improving the prognosis of PC is one of the important research topics of clinical scholars over the years.

This study aimed to evaluate individual clinicopathological characteristics, laboratory test, and operation that can be used to predict survival in patients with PC. We hope that the identification of these factors will help in the development of a treatment plan and ultimately improve the survival of patients with PC.

## Materials and Methods

### General information

Clinicopathological data of 625 patients with PC who were treated in the Department of General Surgery, Shengjing Hospital of China Medical University, from January 2013 to December 2017 were retrospectively collected. Patients with pancreatic metastasis of other cancers, coexisting with other cancers, and perioperative death were excluded. Among the included patients, 341 were male and 284 were female, with a male-to-female ratio of 1.2:1. The patients were aged 27–88 years, with an average of 60.65 years. Of the total of 625 patients, 517 had pathological diagnosis (409 patients had postoperative pathology, 8 patients underwent endoscopic ultrasound-guided needle biopsy), 108 patients were diagnosed according to clinical symptoms and signs, imaging (abdominal ultrasound, CT, MRI, ERCP and PET-CT), and tumor markers (carbohydrate antigen [CA]199 and carcinoembryonic antigen [CEA]). There were 413 patients (66.1%) with pancreatic head cancer [PHC], 207 (33.1%) with pancreatic body/tail cancer [PBTC], and 5 (0.8%) with total PC. Moreover, 423 (67.7%) patients did not have lymph node metastasis, while 202 (32.3%) had lymph node metastasis. Of the 163 patients with distant metastasis, 111 had single-organ metastasis (5 with mesenteric metastasis, 10 with greater/lesser omentum metastasis, 7 with lung metastasis, 3 with colon metastasis, 8 with single abdominal/pelvic metastasis, 2 with spleen metastasis, 4 with kidney/adrenal metastasis, 1 with stomach metastasis, 1 with lumbar metastasis, 1 with retroperitoneal metastasis, 69 with hepatic metastasis), and 52 had multiple-organ metastasis. Of the patients with multiple-organ metastasis, 4 were with liver co-existing mesenteric metastasis, 7 with liver co-existing omentum metastasis, 4 with liver co-existing multiple abdominal metastasis, 1 with liver co-existing colon metastasis, 10 with mesentery co-existing omentum metastasis, 4 with greater omentum co-existing lesser omentum metastasis, 11 with greater omentum co-existing peritoneum metastasis, 4 with peritoneal co-existing retroperitoneal metastasis, 5 with abdominal co-existing pelvic metastasis, 1 with kidney co-existing adrenal metastasis, and 1 with stomach co-existing spleen metastasis. Liver metastasis was noted in 85 patients (52.1%). Of the 69 PC patients with liver metastases only, 52 had resectable or borderline resectable pancreatic tumor (28 with resectable, 24 with borderline resectable), while 17 had locally advanced (unresectable) pancreatic tumor. According to the 8th American Joint Committee on Cancer staging system, of 625 patients, 196 (31.4%) had stage I, 143 (22.9%) had stage II, 123 (19.7%) had stage III, and 163 (26.1%) had stage IV.

### Therapeutic methods

For patients with rescetable or borderline rescetable PC, radical surgery is performed. Specific palliative measures are performed for patients with advanced PC characterized by biliary or gastrointestinal obstruction, severe abdominal pain [11]. Non-operation if the patient and family give up treatment.

Of 625 patients with PC, 365 (58.4%) underwent radical resection (244 underwent radical pancreatoduodenectomy (115 underwent total mesopancreas excision with pancreaticoduodenectomy, 129 underwent pancreatoduodenectomy), 118 underwent radical distal pancreatectomy, 3 underwent total pancreatectomy), 81 (13.0%) underwent palliative operation (9 underwent celiac plexus resection, 17 underwent gastrojejunostomy, 33 underwent cholangiojejunostomy, 12 underwent gastrojejunostomy and cholangiojejunostomy, 5 underwent endoscopic biliary stent implantation, and 5 underwent percutaneous transhepatic cholangial drainage), and 179 (28.6%) did not undergo surgical treatment. A total of 56 patients (9%) received postoperative adjuvant chemotherapy.

According to the consensus developed by the international Pancreatic Fistula Study Group in 2016 [12], among the 365 undergoing radical resection, 344 cases (94.2%) without postoperative pancreatic fistula (POPF), 21 cases (5.8%) with POPF (19 cases of grade B, 2 cases of grade C).

For the 52 resectable or borderline resectable PC patients with hepatic metastasis only, surgical options depend on preoperative, intraoperative integrated assessment and the wish of family. Among the 52 cases, 19 (36.5%) underwent radical resection (synchronous resection of hepatopancreatic lesions), 13 (25%) underwent palliative bypass operation, 20 (38.5%) did not undergo operation.

### Follow-up

All patients were followed from the diagnosis of PC to March 7, 2019, by telephone, short message, and review of patient medical records. Among them, 569 patients were followed effectively, with a follow-up rate of 91%. The follow-up period was 1–75 months. Survival time was measured in months. Patients who died of PC during the follow-up period were considered as having complete data. Those who survived or lost connection at the deadline of the follow-up period were treated as having censored data.

### Statistical analysis

SPSS 23.0 statistical software was used in the statistical analysis. If the continuous variables are normally distributed, they are presented as mean ± standard deviation, and T-test was conducted; otherwise, it was presented as median, and the U-test was adopted. T-test or U-test was adopted to compare the mean values of two subgroups (PHC and PBTC). Some variables (e.g., smoking history, diabetes history, CEA level, albumin level, neutrophil-lymphocyte ratio (NLR), degree of weight loss, peripancreatic invasion) were converted into categorical variables or ranked data. The chi-square test (Fisher’s exact test) or Pearson chi-square test was used to compare the distribution of the two subgroups.

The survival rate and median overall survival (mOS) time were calculated using the Kaplan-Meier method; then, survival curves were drawn. The log-rank test (univariate analysis) was used to compare the difference among the groups. Factors with statistical significance in the univariate analysis were included in the Cox regression model for multivariate analysis. A *P*-value <0.05 was considered statistically significant.

## Results

### Survival situation

**Fig. 1:**
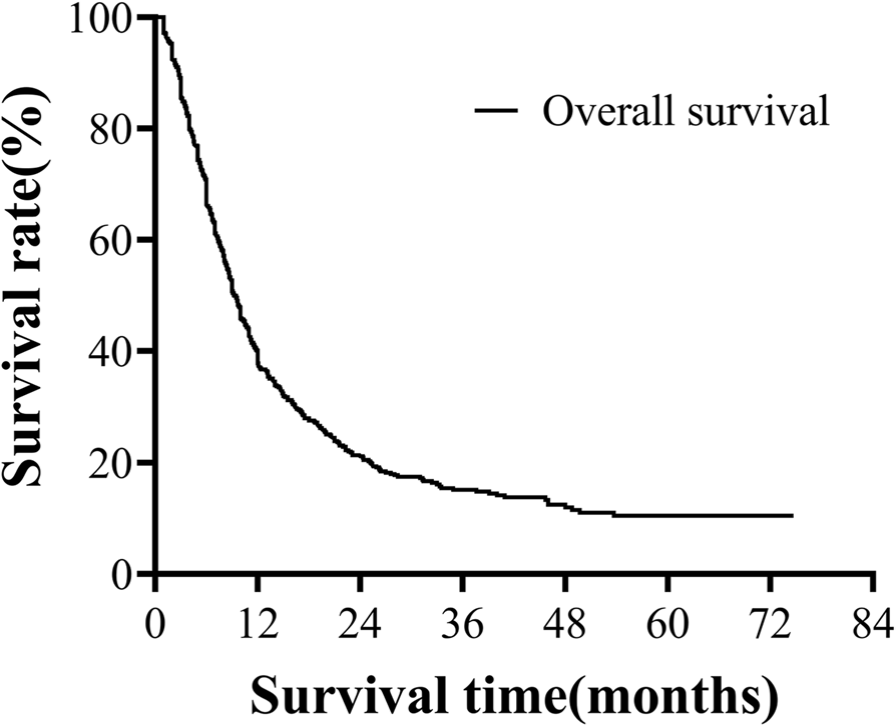
Overall survival of 625 patients with PC. The overall 1-, 3-, and 5-year survival rates of 625 patients with PC were 37.8%, 15.1%, and 10.5%, respectively. The mOS was 9.3 months (95% CI, 8.5–10.1).

At the end of the follow-up, a total of 478 (76%) patients had died. The overall 1-, 3-, and 5-year survival rates of 625 patients with PC were 37.8%, 15.1%, and 10.5%, respectively. The mOS was 9.3 months (95% CI, 8.5–10.1). The overall 1-, 3-, and 5-year survival rates of patients undergoing radical resection were 51.0%, 19.7%, and 12.4%, respectively, and the mOS was 12.3 months (95% CI, 10.3–14.3). The overall 1-, 3-, and 5-year survival rates of patients undergoing palliative operation were 18.9%, 9.3%, and 0%, respectively, and the mOS was 6.0 months (95% CI, 5.2–6.8). The overall 1-, 3-, and 5-year survival rates of patients who did not undergo surgery were 18.4%, 8.5%, and 0%, respectively, and the mOS was 6.0 months (95% CI, 5.3–6.7). The survival curve of 625 patients with PC is shown in Fig. 1.

### Analysis of prognostic factors in PC group

Univariate analysis showed that age, baseline CA199 level, CEA level, total bilirubin level, albumin level, NLR, weight loss, peripancreatic invasion, operative procedure, tumor size, lymph node metastasis, hepatic metastasis, number of distant organ metastasis, and postoperative adjuvant chemotherapy were the relevant factors affecting the prognosis of the PC group (*P*<0.05).

**Fig. 2:**
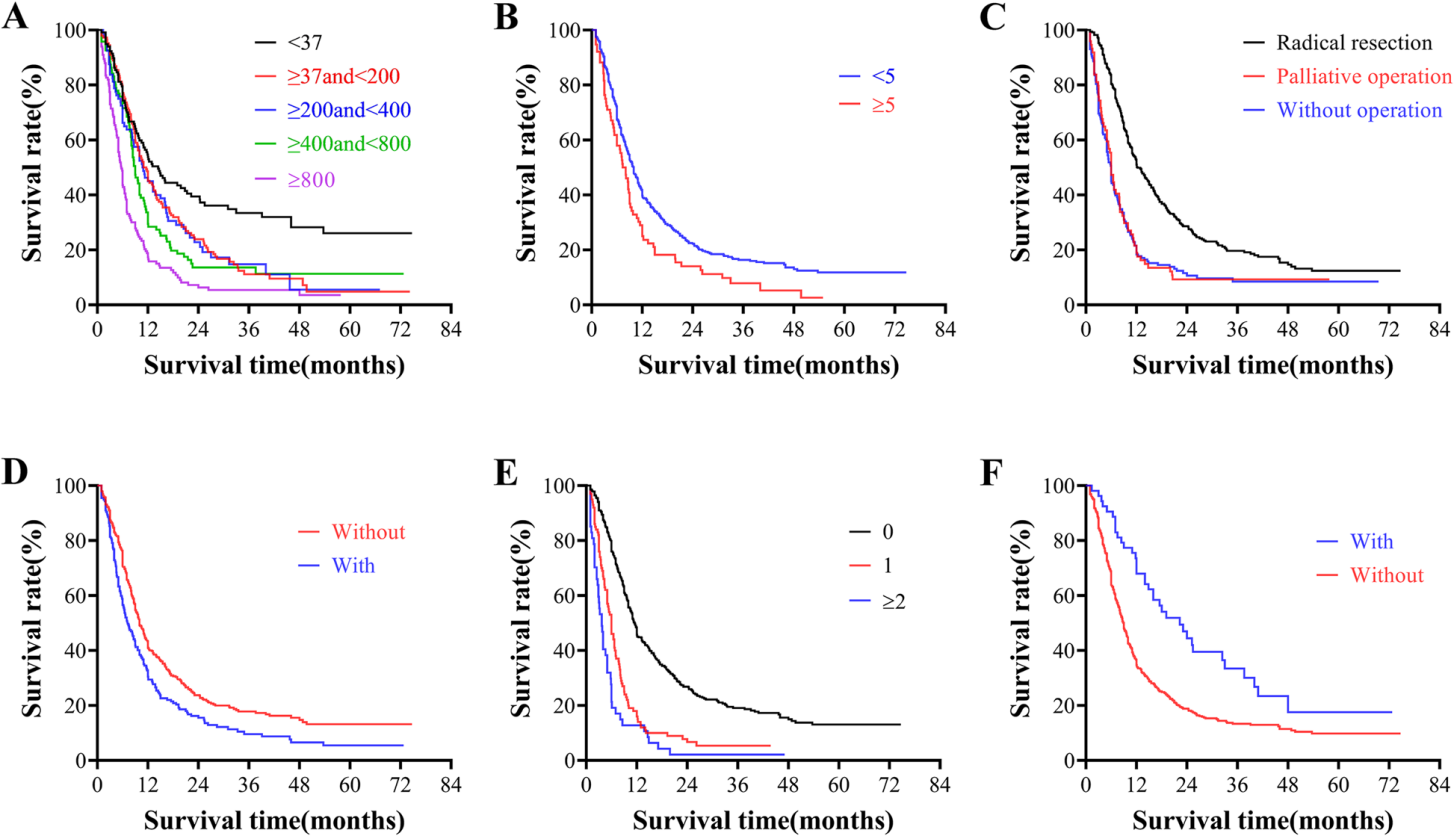
Comparison of survival of patients with different clinical factors. **A** Comparison of survival of patients with different CA199 levels. Survival was significantly worse with increased CA199 levels (*P*<0.05). **B** Comparison of survival of patients with NLR <5 or NLR ≥5. Survival was significantly worse in patients with NLR ≥5 (*P*<0.05). **C** Comparison of survival of patients undergoing different operative procedures. Patients undergoing palliative operation did not have significantly different survival from patients who did not undergo surgery (*P*>0.05). Patients undergoing radical resection had significantly longer survival time than patients who did not undergo radical resection (*P*<0.05). **D** Comparison of survival in patients with or without lymph node metastasis. Survival was significantly worse in patients with lymph node metastasis (*P*<0.05). **E** Comparison of survival in patients with different numbers of distal organ metastasis. Survival was significantly worse with the increase in the number of distal organ metastasis (*P*<0.05). **F** Comparison of survival in patients with or without postoperative adjuvant chemotherapy. Survival was significantly worse in patients without postoperative adjuvant chemotherapy (P<0.05).

Significant factors in the univariate analysis were included in the Cox risk regression model for multivariate analysis, and the results showed that baseline CA199 level, NLR, operative procedure, lymph node metastasis, number of distant organ metastasis, and postoperative adjuvant chemotherapy were independent prognostic factors in the PC group (*P*<0.05) (Table 1, Fig. 2).

**Table 1:** Univariate and Multivariate analysis of prognostic factors for PC

Factors	n(%)	Univariate analysis			Multivariate analysis		
		mOS(95%CI)	χ^**2**^	***p***	B	HR(95%CI)	***P***
Age (years)							
≤40	17 (2.7)	7.4 (4.1-10.7)	13.178	**0.010**		Reference	
41-50	72 (11.5)	12.0 (9.9-14.1)			-0.154	0.857 (0.461-1.594)	0.626
51-60	215 (34.4)	8.7 (7.5-9.9)			0.128	1.136 (0.638-2.024)	0.664
61-70	224 (35.8)	10.3 (8.8-11.8)			0.125	1.133 (0.637-2.016)	0.671
≥71	97 (15.5)	7.3 (5.4-9.2)			0.273	1.314 (0.719-2.401)	0.375
Sex							
Male	341 (54.6)	8.6 (7.5-9.7)	2.412	0.120	-	-	-
Female	284 (45.4)	10.5 (9.2-11.8)			-	-	-
Pain on the back and loin							
No	463 (74.1)	9.5 (8.4-10.6)	0.094	0.759	-	-	-
Yes	162 (25.9)	9.1 (7.4-10.8)			-	-	-
Smoking history							
No	396 (63.4)	9.8 (8.8-10.8)	1.243	0.265	-	-	-
Yes	229 (36.6)	8.5 (7.1-9.9)			-	-	-
Diabetes history							
No	352 (43.7)	9.0 (8.0-10.0)	0.446	0.504	-	-	-
Yes	273 (56.3)	9.6 (8.1-11.1)			-	-	-
CA199(U/ml)							
<37	129 (20.6)	14.5 (10.9-18.1)	67.567	**0.000**		Reference	
≥37and<200	159 (25.4)	11.0 (9.1-12.9)			0.282	1.326 (0.984-1.788)	0.064
≥200and<400	89 (14.2)	10.7 (8.7-12.7)			0.399	1.490 (1.057-2.099)	**0.023**
≥400and<800	101 (16.2)	9.0 (7.9-10.1)			0.474	1.606 (1.157-2.229)	**0.005**
≥800	147 (23.5)	5.7 (5.0-6.4)			0.767	2.153 (1.558-2.975)	**0.000**
CEA (ng/ml)							
<5	400 (64.0)	11.0 (9.9-12.1)	22.771	**0.000**		Reference	
≥5	225 (36.0)	6.7 (6.0-7.4)			0.187	1.205 (0.983-1.478)	0.073
Albumin(g/L)							
<35	76 (12.2)	6.7 (4.5-8.9)	5.128	**0.024**		Reference	
≥35	549 (87.8)	9.7 (8.8-10.6)			-0.137	0.872 (0.643-1.183)	0.379
Total bilirubin (umol/l)							
<22	342 (54.7)	9.0 (8.0-10.0)	8.922	**0.030**		Reference	
≥22and<100	74 (11.8)	7.0 (4.4-9.6)			0.262	1.300 (0.950-1.799)	0.102
≥100and<200	97 (15.5)	10.7 (8.2-13.2)			-0.060	0.942 (0.702-1.264)	0.689
≥200	112 (17.9)	11.3 (8.9-13.7)			0.031	1.032 (0.767-1.388)	0.836
Peripancreatic invasion							
No	242 (38.7)	13.8 (11.3-16.3)	32.275	**0.000**		Reference	
Yes	383 (61.3)	7.4 (6.5-8.3)			0.207	1.230 (0.995-1.519)	0.055
Degree of weight loss (kg)							
<5	358 (57.3)	10.7 (9.6-11.8)	7.755	**0.000**		Reference	
≥5	267 (42.7)	7.8 (6.8-8.8)			0.162	1.176 (0.969-1.427)	0.102
NLR^a^							
<2	135 (21.6)	8.7 (6.9-10.5)	1.765	0.184	-	-	-
≥2	490 (87.4)	9.6 (8.6-10.6)			-	-	-
NLR^b^							
<3	307 (49.1)	9.6 (8.2-11.0)	0.050	0.823	-	-	-
≥3	318 (50.9)	9.1 (7.8-10.4)			-	-	-
NLR^c^							
<4	375 (60.0)	9.7 (8.6-10.8)	1.264	0.261	-	-	-
≥4	250 (40.0)	9.0 (8.0-10.0)			-	-	-
NLR^d^							
<5	545 (87.2)	10.0 (9.1-10.9)	8.602	**0.003**		Reference	
≥5	80 (12.8)	7.4 (5.5-9.3)			0.296	1.344 (1.021-1.769)	**0.035**
Operative procedure							
Without operation	179 (28.6)	6.0 (5.3-6.7)	65.658	**0.000**		Reference	
Palliative operation	81 (13.0)	6.0 (5.2-6.8)			-0.050	0.951 (0.688-1.316)	0.762
Radical resection	365 (58.4)	12.3 (10.3-14.3)			-0.310	0.734 (0.573-0.940)	**0.014**
Tumor location							
Head	413 (66.1)	9.9 (8.7-11.2)	4.484	0.106	-	-	-
Body/tail	207 (33.1)	8.4 (6.7-10.1)			-	-	-
All	5 (0.8)	11.0 (8.9-13.1)			-	-	-
Tumor size (cm)							
d<2	36 (5.8)	12.0 (2.3-21.7)	9.756	**0.000**		Reference	
2≤d<4	314 (50.2)	10.4 (9.2-11.6)			-0.229	0.796 (0.527-1.201)	0.277
d≥4	275 (44.0)	8.2 (7.0-9.4)			-0.035	0.966 (0.630-1.480)	0.873
Hepatic metastasis							
No	540 (86.4)	10.7 (9.7-11.7)	82.708	**0.000**		Reference	
Yes	85 (13.6)	4.5 (3.3-5.7)			0.190	1.209 (0.850-1.720)	0.290
Lymph node metastasis							
No	423 (67.7)	10.0 (9.0-11.0)		**0.000**		Reference	
Yes	202 (32.3)	7.2 (5.7-8.7)			0.395	1.485 (1.218-1.810)	**0.000**
Number of distant organ metastasis							
0	462 (73.9)	11.5 (10.6-12.4)	112.810	**0.000**		Reference	
1	111 (17.8)	6.0 (4.9-7.1)			0.473	1.604 (1.170-2.199)	**0.003**
≥2	52 (8.3)	3.7 (2.8-4.6)			0.708	2.030 (1.347-3.062)	**0.001**
Adjuvant chemotherapy							
No	569 (91.0)	8.9 (8.1-9.7)	16.559	**0.000**		Reference	
Yes	56 (9.0)	22.3 (14.7-29.9)			-0.701	2.015 (1.412-2.877)	**0.000**

### Comparison of clinicopathological data between the PHC subgroup and PBTC subgroup

To examine whether the location of the tumor was associated with the clinicopathological features, we compared the PHC and PBTC. There was no statistically significant difference in age, sex, smoking and diabetes history, baseline CA199 level, CEA level, NLR, weight loss, lymph node metastasis, and postoperative adjuvant chemotherapy between PHC and PBTC subgroups (*P*>0.05). The diameter of PBTC was significantly larger than that of PHC (4.4 [1.2–11.2] cm vs. 3.1 [1.0–9.0] cm, *P*<0.01). Pain on the back and loin, peripancreatic invasion, and hepatic metastasis were more common in the PBTC subgroup (*P*<0.01). Serum albumin level < 35 g/L was more common in the PHC subgroup (*P*<0.01). The baseline total bilirubin level in the PHC subgroup was significantly higher than that in the PBTC subgroup (97.9 [0.92–1221.8] umol/L vs. 10.4 [2.3–405.7] umol/L, *P*<0.01). Nonsurgical treatment was more common in the PBTC subgroup (*P*<0.01). The number of distant organ metastasis in the PBTC subgroup was higher than that in the PHC subgroup (*P*<0.01). In the PHC group, the mOS was 9.9 months (95% CI, 8.7–11.2), and the 1-, 3-, and 5-year survival rates were 39.9%, 16.9%, and 11.3%, respectively. In the PBTC group, the mOS was 8.4 months (95% CI, 6.7–10.1), and the 1-, 3-, and 5-year survival rates were 33.3%, 11.9%, and 9.1%, respectively. The difference was statistically significant (*P*<0.05) (Table 2).

**Table 2:** Comparison of clinicopathological data and survival between the PHC subgroup and PBTC subgroup

Characteristics	Tumor location		***P***
	Head	Body/tail	
mOS(95%CI)	9.9 (8.7-11.2)	8.4 (6.7-10.1)	0.041***
Age (years)	60.47±9.76	60.94±9.63	0.568*
Sex Male:Female	224:189	115:92	0.798**
Pain on the back and loin No:Yes	334:79	128:79	0.000**
Somking history No:Yes	254:159	139:68	0.098**
Diabetes history No:Yes	233:180	119:88	0.434**
CA199(U/ml)	258.7 (0.5-37192)	195 (0.5-327524)	0.336^##^
CEA (ng/ml) <5:≥5	272:141	127:80	0.287**
Total bilirubin (umol/l)	97.9 (0.92-1221.8)	10.4 (2.3-405.7)	0.000^##^
Albumin(g/l) <35:≥35	61:352	15:192	0.006**
NLR <5:≥5	361:52	180:27	0.899**
Peripancreatic invasion No:Yes	194:219	46:161	0.000**
Weight loss (kg) <5:≥5	234:179	123:84	0.547**
Operative procedure Wo:Po:Rr	99:70:244	79:10:118	0.000^#^
Tumor size (cm)	3.1 (1.0-9.0)	4.4 (1.2-11.2)	0.000^##^
Lymph node metastasis No:Yes	277:136	141:66	0.856**
Hepatic metastasis No:Yes	370:43	166:41	0.020**
Number of distant organ metastatic 0:1:≥2	339:60:14	119:51:37	0.000^#^
Adjuvant chemotherapy No:Yes	376:37	188:19	0.518**

P was examined by the ^#^Pearson chi-square test or ^##^Mann-Whitney U test or *Student’s t-test or **chi-square test (Fisher’s exact test) or ***log-rank test for univariate survival analysis

Numbers in parentheses are the ranges of values

*NLR* Neutrophil-lymphocyte ratio

*Wo* Without operation, *Po* Palliative operation, *Rr* Radical resection

### Prognostic factors analysis of PHC subgroup and PBTC subgroup

Univariate and multivariate analyses were respectively performed on 413 patients with PHC and 207 patients with PBTC. Univariate analysis showed that age, baseline CA199 level, CEA level, albumin level, peripancreatic invasion, degree of weight loss, NLR, operative procedure, lymph node metastasis, hepatic metastasis, the number of distant organ metastasis and postoperative adjuvant chemotherapy were the relevant factors affecting the prognosis of PHC subgroup (*P*<0.05). Multivariate analysis showed that degree of weight loss, baseline CA199 level, NLR, operative procedure, the number of distant organ metastasis, lymph node metastasis, and postoperative adjuvant chemotherapy were independent prognostic factors in the PHC subgroup (*P*<0.05), as shown in Table 3.

**Table 3:** Univariate and Multivariate analysis of prognostic factors for PHC subgroup

Factors	n(%)	Univariate analysis			Multivariate analysis		
		mOS(95%CI)	χ^**2**^	***p***	B	HR(95%CI)	***P***
Age (years)							
≤40	9 (2.2)	7.4 (5.0-9.8)	11.211	**0.024**		Reference	
41-50	53 (12.8)	14.5 (9.3-19.6)			-0.009	0.991 (0.406-2.420)	0.984
51-60	148 (35.8)	8.6 (7.6-9.6)			0.066	1.068 (0.456-2.501)	0.880
61-70	142 (34.4)	11.3 (9.9-12.6)			0.148	1.159 (0.496-2.707)	0.733
≥71	61 (14.8)	6.7 (3.8-9.6)			0.298	1.347 (0.562-3.233)	0.504
Sex							
Male	189 (45.8)	9.0 (7.8-10.2)	1.080	0.299	-	-	-
Female	224 (54.2)	11.0 (9.2-12.8)			-	-	-
Pain on the back and loin							
No	334 (80.9)	10.0 (8.4-11.6)	0.232	0.630	-	-	-
Yes	79 (19.1)	9.3 (6.8-11.8)			-	-	-
Smoking history							
No	254 (61.5)	10.0 (8.6-11.4)	0.292	0.589	-	-	-
Yes	159 (38.5)	9.7 (8.6-11.4)			-	-	-
Diabetes history							
No	180 (43.6)	9.2 (7.7-10.7)	0.054	0.817	-	-	-
Yes	223 (56.4)	10.0 (8.1-11.9)			-	-	-
CA199(U/ml)							
<37	78 (18.9)	15.5 (9.2-21.8)	33.008	**0.000**		Reference	
≥37and<200	104 (25.2)	10.7 (8.1-13.3)			0.485	1.624 (1.122-2.351)	**0.010**
≥200and<400	65 (15.7)	10.7 (8.1-13.3)			0.549	1.732 (1.142-2.628)	**0.010**
≥400and<800	72 (17.4)	9.6 (8.0-11.2)			0.498	1.646 (1.100-2.462)	**0.015**
≥800	94 (22.8)	6.4 (5.3-7.5)			0.898	2.454 (1.626-3.702)	**0.000**
CEA (ng/ml)							
<5	272 (65.9)	11.1 (9.7-12.5)	8.044	**0.000**		Reference	
≥5	141 (34.1)	7.1 (5.6-8.6)			0.075	1.078 (0.834-1.393)	0.566
Albumin(g/L)							
<35	61 (14.8)	6.7 (4.1-9.3)	5.050	**0.025**		Reference	
≥35	352 (85.2)	10.3 (9.0-11.6)			-0.181	0.835 (0.597-1.166)	0.290
Total bilirubin (umol/l)							
<22	155 (37.5)	9.0 (7.2-10.8)	6.096	0.107	-	-	-
≥22and<100	53 (12.8)	8.0 (5.6-10.4)			-	-	-
≥100and<200	95 (23.0)	10.7 (8.4-13.0)			-	-	-
≥200	110 (26.6)	11.3 (8.8-13.8)			-	-	-
Peripancreatic invasion							
No	194 (47.0)	14.0 (11.2-16.8)	17.015	**0.000**		Reference	
Yes	219 (53.0)	7.4 (6.3-8.3)			0.154	1.167 (0.912-1.493)	0.219
Degree of weight loss (kg)							
<5	234 (56.7)	11.6 (10.5-12.7)	8.223	**0.000**		Reference	
≥5	179 (43.3)	8.0 (6.8-9.2)			0.296	1.345 (1.060-1.707)	**0.015**
NLR^a^							
<2	98 (23.7)	8.1 (6.1-10.1)	3.362	0.067	-	-	-
≥2	315 (76.3)	10.7 (9.3-12.1)			-	-	-
NLR^b^							
<3	206 (49.9)	9.6 (7.9-12.3)	0.079	0.779	-	-	-
≥3	207 (50.1)	10.0 (8.3-11.7)			-	-	-
NLR^c^							
<4	248 (60.0)	10.0 (8.4-11.6)	1.433	0.231	-	-	-
≥4	165 (40.0)	9.1 (7.2-11.0)			-	-	-
NLR^d^							
<5	361 (87.4)	10.0 (8.5-11.5)	4.660	**0.031**		Reference	
≥5	52 (12.6)	8.6 (7.2-10.0)			0.206	1.229 (0.878-1.721)	0.229
Operative procedure							
Without operation	99 (24.0)	6.0 (5.1-6.9)	46.087	**0.000**		Reference	
Palliative operation	70 (16.9)	6.5 (4.8-8.2)			-0.300	0.741 (0.507-1.083)	0.121
Radical resection	244 (59.1)	14.5 (11.7-17.3)			-0.583	0.558 (0.409-0.761)	**0.000**
Tumor size (cm)							
d<2	32 (7.7)	15.5 (3.8-27.2)	4.244	0.120	-	-	-
2≤d<4	238 (57.6)	11.0 (9.4-12.6)			-	-	-
d≥4	143 (34.6)	8.4 (6.8-10.0)			-	-	-
Hepatic metastasis							
No	370 (89.6)	11.1 (10.0-12.2)	42.481	**0.000**		Reference	
Yes	43 (10.4)	5.0 (3.6-6.4)			-0.131	0.877 (0.514-1.497)	0.631
Lymph node metastasis							
No	136 (32.9)	11.3 (10.1-12.5)	18.045	**0.000**		Reference	
Yes	277 (67.1)	7.2 (5.5-8.9)			0.527	1.694 (1.328-2.161)	**0.000**
Number of distant organ metastasis							
0	339 (82.1)	11.6 (10.0-13.2)	74.641	**0.000**		Reference	
1	60 (14.5)	6.0 (4.5-7.5)			0.707	2.028 (1.286-3.199)	**0.002**
≥2	14 (3.4)	2.9 (1.0-5.1)			0.791	2.205 (1.033-4.706)	**0.041**
Adjuvant chemotherapy							
No	376 (91.0)	9.0 (8.0-10.0)	10.973	**0.000**		Reference	
Yes	37 (9.0)	25.4 (9.1-41.7)			-0.513	0.599 (0.384-0.933)	**0.023**

Univariate analysis showed that baseline CA199 level, CEA level, total bilirubin level, peripancreatic invasion, NLR, operative procedure, lymph node metastasis, hepatic metastasis, number of distant organ metastasis, and postoperative adjuvant chemotherapy were the relevant factors affecting the prognosis of the PBTC subgroup (*P*<0.05). Multivariate analysis showed that baseline CA199 level, CEA level, total bilirubin level, NLR, peripancreatic invasion, postoperative adjuvant chemotherapy, and number of distant organ metastasis were independent prognostic factors in the PBTC subgroup (*P*<0.05) (Table 4).

**Table 4:** Univariate and Multivariate analysis of prognostic factors for PBTC subgroup

Factors	n(%)	Univariate analysis			Multivariate analysis		
		mOS(95%CI)	χ^**2**^	***p***	B	HR(95%CI)	***P***
Age (years)							
≤40	8 (3.9)	5.0 (0.0-25.7)	2.455	0.653	-	-	-
41-50	19 (9.2)	8.2 (3.7-12.7)			-	-	-
51-60	66 (31.9)	9.0 (5.4-12.6)			-	-	-
61-70	78 (37.7)	8.2 (6.2-10.2)			-	-	-
≥71	36 (17.4)	8.3 (6.3-10.3)			-	-	-
Sex							
Male	115 (55.6)	7.0 (5.6-8.4)	1.589	0.208	-	-	-
Female	92 (44.4)	9.7 (8.3-11.1)			-	-	-
Pain on the back and loin							
No	128 (61.8)	8.2 (6.0-10.4)	0.909	0.340	-	-	-
Yes	79 (38.2)	8.7 (6.7-10.8)			-	-	-
Smoking history							
No	139 (67.1)	9.6 (8.0-11.2)	1.807	0.179	-	-	-
Yes	68 (32.9)	7.0 (5.7-8.3)			-	-	-
Diabetes history							
No	119 (57.5)	9.0 (7.6-10.4)	0.864	0.353	-	-	-
Yes	88 (42.5)	7.0 (5.0-9.0)			-	-	-
CA199(U/ml)							
<37	50 (24.2)	10.5 (6.1-14.9)	41.985	**0.000**		Reference	
≥37and<200	54 (26.1)	12.0 (9.4-14.6)			0.056	1.058 (0.647-1.729)	0.823
≥200and<400	24 (11.6)	10.6 (8.0-13.2)			0.175	1.191 (0.642-2.211)	0.579
≥400and<800	28 (13.5)	7.6 (4.0-11.2)			0.757	2.131 (1.162-3.909)	**0.015**
≥800	51 (24.6)	5.0 (4.1-5.9)			0.813	2.255 (1.305-3.897)	**0.004**
CEA (ng/ml)							
<5	127 (61.4)	10.6 (8.8-12.4)	17.747	**0.000**		Reference	
≥5	80 (38.6)	6.0 (5.4-6.7)			0.508	1.661 (1.152-2.397)	0.007
Albumin(g/L)							
<35	15 (7.2)	3.2 (0.0-7.8)	1.494	0.222	-	-	-
≥35	192 (92.8)	8.7 (7.1-10.3)			-	-	-
Total bilirubin (umol/l)							
<22	184 (88.9)	8.8 (7.3-10.3)	9.531	**0.023**		Reference	
≥22and<100	21 (10.1)	5.4 (5.0-5.8)			0.620	1.858 (1.070-3.229)	**0.028**
≥100and<200	1 (0.5)	-			-1.082	0.339 (0.038-3.042)	0.334
≥200	1 (0.5)	-			1.780	5.927 (0.752-46.746)	0.091
Peripancreatic invasion							
No	46 (22.2)	13.4 (6.5-20.3)	10.891	**0.000**		Reference	
Yes	161 (77.8)	7.5 (6.1-8.9)			0.501	1.650 (1.077-2.529)	**0.021**
Degree of weight loss (kg)							
<5	123 (59.4)	9.0 (7.5-10.5)	0.567	0.452	-	-	-
≥5	84 (40.6)	7.0 (5.5-8.5)			-	-	**-**
NLR^a^							
<2	36 (17.4)	9.0 (5.8-12.2)	0.028	0.868	-	-	-
≥2	171 (82.6)	8.3 (6.7-9.9)			-	-	-
NLR^b^							
<3	99 (47.8)	9.0 (6.7-11.3)	0.000	0.993	-	-	-
≥3	108 (52.2)	8.2 (6.6-9.8)			-	-	-
NLR^c^							
<4	124 (59.9)	9.0 (7.3-10.7)	0.190	0.663	-	-	-
≥4	83 (40.1)	7.5 (5.8-9.2)			-	-	-
NLR^d^							
<5	180 (87.0)	9.0 (7.5-10.5)	5.990	**0.014**		Reference	
≥5	27 (13.0)	3.4 (0.0-8.1)			0.500	1.649 (1.009-2.696)	**0.046**
Operative procedure							
Without operation	79 (38.2)	5.8 (4.9-6.7)	21.185	**0.000**		Reference	
Palliative operation	10 (4.8)	2.0 (0.7-3.3)			0.615	1.850 (0.817-4.189)	0.140
Radical resection	118 (57.0)	11.0 (8.4-13.6)			0.153	1.166 (0.785-1.731)	0.447
Tumor size (cm)							
d<2	4 (1.9)	6.0 (0.0-14.1)	2.697	0.260	-	-	-
2≤d<4	74 (35.7)	9.0 (6.1-11.9)			-	-	-
d≥4	129 (62.3)	8.1 (6.6-9.6)			-	-	-
Hepatic metastasis							
No	41 (19.8)	10.0 (8.7-11.3)	30.997	**0.000**		Reference	
Yes	166 (80.2)	4.5 (2.4-6.6)			0.462	1.588 (0.966-2.610)	0.068
Lymph node metastasis							
No	141 (68.1)	7.1 (4.0-20.2)	0.091	0.763	-	-	-
Yes	66 (31.9)	8.7 (7.2-10.2)			-	-	**-**
Number of distant organ metastasis							
0	119 (57.5)	11.0 (9.3-12.7)	36.997	**0.000**		Reference	
1	51 (24.6)	6.0 (4.5-7.5)			0.468	1.597 (1.009-2.527)	**0.046**
≥2	37 (17.9)	4.0 (2.4-5.6)			0.771	2.163 (1.268-3.690)	**0.005**
Adjuvant chemotherapy							
No	188 (90.8)	7.7 (6.2-9.2)	5.857	**0.016**		Reference	
Yes	19 (9.2)	17.5 (7.7-27.3)			-1.077	0.340 (0.182-0.638)	**0.001**

*NLR* Neutrophil-lymphocyte ratio, *HR* Hazard ratio, *CI* Confidence interval, *mOS* Median overall survival

*p*: Log Rank Mantel–Cox test; P: Cox proportional hazards model

### Survival analysis and comparison of different operation procedures in resectable or borderline resectable PC patients with hepatic metastasis only

Survival analysis was performed on 52 resectable or borderline resectable PC patients with hepatic metastasis only. The overall 1-, 3-, and 5-year survival rates of the 52 patients were 12.8%, 6.4%, and 0%, respectively, and the mOS was 5.2 months (95% CI, 3.8–6.6). The overall 1-, 3-, and 5-year survival rates of patients undergoing radical resection (synchronous resection of pancreatic and hepatic lesions) were 22.2%, 11.1%, and 0%, respectively, and the mOS was 4.4 months (95% CI, 1–8.5). The overall 1-, 3-, and 5-year survival rates of patients who did palliative bypass operation were 9.1%, 0%, and 0%, respectively, and the mOS was 6.0 months (95% CI, 3.1–8.9). The overall 1-, 3-, and 5-year survival rates of patients who did not undergo surgical were 5.6%, 0%, and 0%, respectively, and the mOS was 5.0 months (95% CI, 2.1–7.9). There was no statistically significant difference between the different operative procedures (*P*>0.05).

### Survival analysis of patients with or without POPF after radical resection

Survival analysis was performed on 365 underwent radical resection. The overall 1-, 3-, and 5-year survival rates of patients without POPF were 52.5%, 20.5%, and 12.9%, respectively, and the mOS was 13.2 months (95% CI, 11.5–15.0). The overall 1-, 3-, and 5-year survival rates of patients with POPF were 28.6%, 5.7%, and 0%, respectively, and the mOS was 8.4 months (95% CI, 3.8–12.0). The difference was statistically significant (*P*<0.05).

### Survival analysis and comparison of different operation procedures in PHC subgroup underwent radical pancreatoduodenectomy

Survival analysis was performed on 244 underwent radical pancreatoduodenectomy. The overall 1-, 3-, and 5-year survival rates of patients underwent total mesopancreas excision with pancreaticoduodenectomy (TMpE) were 61.5%, 26.3%, and 15.5%, respectively, and the mOS was 17.5 months (95% CI, 15.0–20.0). The overall 1-, 3-, and 5-year survival rates of patients underwent pancreaticoduodenectomy (PD) were 46.2%, 18.6%, and 11.2%, respectively, and the mOS was 11.5 months (95% CI, 9.3–13.7). The difference was no statistically significant (*P*=0.126).

## Discussion

### The relationship between prognosis and general clinical features

Similar to some previous reports [13, 14], we found that sex and age had no effect on the prognosis of patients with PC. The conclusion of whether diabetes affected the prognosis of PC was still inconsistent. A meta-analysis [15] showed that patients with PC with diabetes had shorter survival time and more complications. However, Cheon et al., [16] reported that patients with PC did show a significant difference in survival time regardless of whether they had diabetes, and those with high glycated hemoglobin level had a shorter survival time. In our study, we found that diabetes history was not associated with the prognosis of PC, which still need further detailed study.

It has been known that most patients with malignant tumor exhibit weight loss and decreased albumin levels. In patients with PC, the risk of weight loss and malnutrition was significantly increased due to impaired endocrine and exocrine functions of the pancreas [17]. Studies have found that weight loss [18] and lower albumin levels [19] were associated with high mortality in PC. Sakamoto et al., [20] showed that sarcopenia at the time of recurrence was an independent, unfavorable prognostic factor in patients with recurrent PC. Yamada et al., [21] reported that frailty (defined as a clinical frailty scale score ≥4) was an independent prognostic factor of cancer-specific survival in patients with pancreatic ductal adenocarcinoma who underwent pancreatic resection. Our study found that in the PHC subgroup, patients with weight loss ≥5 kg had significantly shorter mOS than patients with weight loss <5 kg (8.0 months vs. 11.6 months, *P*<0.01). Weight loss ≥5 kg was an independent prognostic factor for PHC. Therefore, clinicians should pay attention to the occurrence of weight loss in patients with PHC. An appropriate nutritional intervention should be performed to correct the cachexia status of patients, improving the prognosis of patients.

Recently, individuals pay more attention to the relationship between inflammatory immune response and tumor prognosis. The state of host immune system has a significant impact on the prognosis of many malignant tumors, including gastrointestinal tumors [22]. Lin et al., [23] revealed that high pretreatment lymphocyte to monocyte ratio (LMR) predicted better overall survival (HR=0.68, 95% CI: 0.58–0.80, *P*<0.001) in patients with PC. Sierzega et al., [24] reported that patients with NLR ≥5 had significantly lower mOS than patients with NLR <5 (12.6 months vs. 25.7 months, *P*<0.01), and the multivariate analysis showed that high NLR was an independent risk factor for poor prognosis in patients with PC. Poor prognosis may be due to changes in the number of neutrophil and lymphocyte subsets, resulting in a decrease in effective antitumor cells and increase in cells involved in immunosuppression. Mowbray et al., [25] reported that patients with high preoperative NLR had poor prognosis. In early studies, the critical value of NLR was from 2 to 5, which may be related to the differences in sample size, population, and treatment method. Based on previous studies, we set the critical value of NLR to 2, 3, 4, and 5. We found that, when the critical value of NLR was set at 5, patients with NLR ≥5 had significantly shorter mOS than patients with NLR <5 (7.4 months vs. 10.0 months, *P*<0.01). In the PBTC subgroup, patients with NLR ≥5 had significantly shorter mOS than patients with NLR <5 (3.4 months vs. 9.0 months, *P*=0.014). Moreover, the multivariate analysis showed that NLR ≥5 was an independent prognostic factor in the PC and PBTC subgroups.

Takamori et al., [26] found that back and loin pain indicated peripancreatic and retroperitoneal nerve invasion and poor prognosis. In our study, although back and loin pain was not associated with the prognosis of PC, we found that back and loin pain was more common in the PBTC subgroup (*P*<0.01). It can be used as a relatively specific clinical manifestation of PBTC. In clinical work, back and loin pain should not be simply diagnosed as neuromuscular or orthopedic disease. We should be alert on the occurrence of PBTC. Patients should undergo abdominal CT or MRI to improve the diagnostic rate of PC. Lee et al., [27] reported that preoperative jaundice is the only independent prognostic factor for PC. Nakata et al., [28] reported that the difference in mOS between the jaundice and nonjaundice groups was significant in resectable cases but not in nonresectable cases. Our results showed that the baseline total bilirubin level was not related to the prognosis of PC but was related to the prognosis of the PBTC subgroup. Furthermore, our data indicated that the increase in the baseline total bilirubin level in the PBTC subgroup was not significant, and the baseline total bilirubin level was >100 umol/L in only a few patients. In the PBTC subgroup, the slight increase in baseline total bilirubin level was probably due to the presence of liver metastasis, which usually occurs in advanced disease [29]. However, we found that liver metastasis was not related to the prognosis of PBTC subgroup, which needs further study.

### Effects of CA199 and CEA levels on prognosis

CA199 is a tumor-associated antigen of the digestive system, which is usually used in preoperative examination of PC. CA199 has the role of intercellular adhesion molecule, and its elevated expression level often indicates the presence of cancer metastasis [30]. The higher the preoperative CA199 level, the greater the risk of distant metastasis and poor prognosis. Hartwig et al., [31] reported that elevated preoperative CA199 levels were related to poor prognosis of PC. CA199 level is not elevated in approximately 10% of patients with PC because Lewis antigen is negative. To date, other tumor markers, such as CEA, should be combined to assist diagnosis [32]. Study [33] have indicated that CA199 and CEA levels are closely associated with lymph node metastasis and tumor progression in PC. In our study, we found higher baseline CA199 level caused shorter mOS; baseline CA199 level was an independent prognostic factor in the PC, PHC, and PBTC subgroups. In addition, baseline CEA level was an independent prognostic factor in the PBTC subgroup. Because of the lack of effective data, we were unable to show the effect of CA199 dynamic changes on postoperative prognosis of patients in this study.

### The effects of tumor location, peripancreatic invasion, lymph node metastasis, hepatic metastasis and the number of distal organ metastasis on prognosis

Our study found that PHC accounted for 66.1% and PBTC accounted for 33.1%, which is similar to a study [34]. They found that the vast majority of PC are in the head, while 20% to 25% are located in the body/tail. Some studies have suggested that the location of pancreatic tumors is a potential determinant of survival [35, 36]. A study [37] found that the survival time of patients with PBTC is shorter than that of patients with PHC. In our study, we found that the mOS of the PHC subgroup was significantly longer than that of the PBTC subgroup (9.9 months vs. 8.4 months, P=0.041). Generally, tumors in the body/tail are found later until they present significant clinical symptoms. At this point, the tumors often infiltrate adjacent organs or vascular structures and possibly metastasize to locoregional lymph nodes via lymphatic vessels or distant organs via hematogenous dissemination [38, 39]. However, tumors located in the head of the pancreas were more likely to compress the common bile duct, resulting in obstructive jaundice, which can be detected promptly [36].

PC has hematogenous and lymph node metastases common with other solid tumors, and its inherent peripancreatic invasion, especially postpancreatic and extrapancreatic plexus invasion, has a high incidence, even in patients who underwent histological radical surgery, and their prognosis is still poor [40]. McKay et al., [41] indicated that lymphatic metastasis is one of the most important factors affecting survival after radical excision of PHC. They found that the 5-year survival rate of patients without lymphatic metastasis was significantly higher than that of patients with lymphatic metastasis. We found that patients with peripancreatic invasion had a significantly shorter mOS than those without peripancreatic invasion in the PC group (7.4 months vs. 13.8 months, *P*<0.01). We also found that peripancreatic invasion was more common in the PBTC subgroup. In the PBTC subgroup, patients with peripancreatic invasion had a significantly shorter mOS than those without peripancreatic invasion (7.5 months vs. 13.4 months, *P*<0.01). Peripancreatic invasion is an independent prognostic factor in the PC and PBTC subgroups. This may be one of the reasons that the prognosis in the PBTC subgroup is worse than that in the PHC subgroup. Our study showed that, in the whole PC group, patients with lymphatic metastasis had a significantly shorter mOS than those without lymphatic metastasis (7.2 months vs. 10.0 months, *P*<0.01). In the PHC subgroup, patients with lymphatic metastasis had a significantly shorter mOS than those without lymphatic metastasis (7.2 months vs. 11.3 months, *P*<0.01). Lymphatic metastasis is an independent prognostic factor in the PC and PHC subgroups.

Wright et al., [42] reported that hepatic metastasis accounts for approximately 70% of cases initially diagnosed as stage IV PC. Our study showed that, among 162 patients with distant metastasis, 85 (52.5%) had hepatic metastasis and the liver was the most common organ of distant metastasis of PC. Moreover, the mOS of patients with hepatic metastasis is low. We also found that hepatic metastasis is more common in the PBTC subgroup. Recently, some studies reported many therapeutic methods and efficacy of PC with hepatic metastasis; however, the conclusions were different, and there is no formal treatment principle. A retrospective analysis on 69 patients with PC with hepatic metastasis who underwent concurrent surgical resection from six European pancreatic centers showed that the mOS of patients undergoing synchronous resection of hepatopancreatic lesions was significantly longer than those without synchronous resection (14 months vs. 7.5 months, *P*<0.01). This benefit was only found in PHC but not in PBTC. Furthermore, the study showed that, due to the limitations of the retrospective study, the conclusion that synchronous hepatopancreatic lesion resection is effective could not be drawn and a prospective study is still needed to confirm this [43]. Shrikhande et al., [44] reported that difference in survival between R0/R1 M1 (liver metastases) and M1 (liver metastases) without any resection (exploration/bypass) was statistically significant (P=0.0384), with a median survival of 11.4 months (95% CI, 7.8–16.5) for the R0/R1 M1 group compared with 5.9 months (95% CI, 5.4–7.6) for the M1 group (liver metastases) without any resection. However, the decision to resect metastatic disease, especially liver metastasis, should be made with great care after a thorough assessment of the overall risk-benefit ratio for the individual patient. Gleisner et al., [45] reported that synchronous resection of hepatopancreatic lesions does not benefit these patients. Frigerio et al., [46] reported that, in some cases of PC with distant solitary metastasis, if the tumor is significantly reduced after a systematic chemotherapy and R0 resection is expected, radical synchronous hepatopancreatic lesion resection is recommended. Performing a hepatectomy for liver metastases of PC, when combined with a pancreas resection, was considered to be a safe operation, and one that might offer prolonged survival for highly selected patients with curative resection of liver metastases [47]. In PC patients with liver metastases only, we used resectable or borderline resectable pancreatic tumors as the research object to analyze whether simultaneous resection surgery can prolong the survival of these patients. Our study showed that there was no statistically significant difference in mOS between patients who underwent synchronous resection of hepatopancreatic lesions and those who did not. There are several limitations of our study that restrict the value of the conclusions. (1) only a relatively small sample size of PC patients only with liver metastases could be identified for our study. And the statistical analyses and inferences were limited. (2) Our study did not assess other important endpoints, such as quality of life after surgical treatment with synchronous resection of hepatopancreatic lesions versus did not any resection. (3) The analysis are retrospective in nature. Therefore, more large-scale randomized controlled studies are required to determine the treatment principle of PC with hepatic metastasis.

In other malignant tumors, such as pulmonary carcinoma, hepatocellular carcinoma, and bladder cancer, multiple-organ metastasis was one of the poor prognostic factors [48–50]. In this study, we found that the number of distant organ metastasis had a certain impact on the prognosis of PC. The prognosis of patients with single-organ metastasis was better than that in patients with multiple-organ metastasis. The larger the number of distant organ metastasis, the shorter the mOS. The number of distant organ metastasis was an independent prognostic factor in the PC, PHC, and PBTC subgroups. We also found that the number of distant organ metastasis in PBTC subgroup is more than that in the PHC subgroup, which may be the reason that PBTC has a poor prognosis than PHC.

### Relationship between prognosis and treatment

Surgical resection is still the most effective treatment for PC. Radical resection is critical to the prognosis of PC [51]. The diagnosis and therapy concept of PC has been transformed into a multidisciplinary team (MDT) mode [11]. Preoperative MDT discussion was conducted to classify PC into resectable PC, borderline resectable PC, locally advanced PC, and PC with distant metastasis by preoperative imaging assessment and determine which patients should undergo surgery. According to different types of PC, appropriate treatment options are selected to maximize clinical benefits. In resectable PC, radical pancreatoduodenectomy, distal pancreatectomy, and total pancreatectomy are feasible. In patients with borderline resectable PC, neoadjuvant therapy is its preferred treatment modality. Pancreatic surgery is performed by institutions that perform several pancreatectomies annually [52]. Tang et al., [53] reported that the survival time of patients with borderline resectable tumors who received neoadjuvant therapy was similar to those with resectable tumors, which was significantly longer than those with unresectable tumors. Most NCCN member institutions [11] recommend that neoadjuvant therapy rather than surgery for patients with borderline resectable tumors. In patients with locally advanced or distant metastasis, stent implantation, gastrojejunostomy, and choledochojejunostomy can be used to relieve digestive tract or biliary tract obstruction, and pathological diagnosis can be obtained as far as possible. We found that the mOS of patients undergoing radical resection and palliative operation and those who did not undergo surgery were 12.3 months, 6.0 months, and 6.0 months, respectively. Multivariate analysis indicated that radical resection was an independent prognostic factor in PC and PHC subgroups. The prognosis of patients undergoing radical resection was significantly better than those without radical resection (*P*<0.05). However, the difference between palliative operation and non-operation is not statistically significant. Therefore, radical resection is extremely important in the prognosis of patients. Palliative surgery plays a role in alleviating the obstructive symptoms and pain, improving the quality of life of patients, but it cannot prolong the patient’s survival.

Crino et al., [54] reported that endoscopic ultrasound-guided fine-needle biopsy (EUS-FNB) demonstrated high diagnostic accuracy in evaluating solid pancreatic lesion (SPLs) independently, meanwhile, in patients with unresectable PC, tissue biopsy samples are the only available histological material. In addition, repeated EUS-FNB after neoadjuvant chemotherapy may detect therapy-induced molecular changes, for example, mutation in KRAS [55, 56]. Therefore, high-quality histological samples obtained by EUS-FNB will provide the basis for individualized treatment of PC [54].

A POPF is considered one of the common complications after pancreatic surgery, with an incidence of 3%-45% [12]. A retrospective analysis [57] showed that pancreatic fistula was an independent risk factor for peritoneal recurrence (HR:3.974; 95% CI:1.345–11.737; P=0.013). Pancreatic fistula was an independent prognostic factor after multivariate analysis (HR:3.257; 95%CI: 1.201–8.828; P=0.020). There is a significant correlation between the occurrence of POPF and higher postoperative mortality. The main reason is that the leaked pancreatic juice erodes the surrounding tissues, causing complications such as secondary intra-abdominal hemorrhage and infection [58, 59]. Our results also show that patients with POPF had a significantly shorter mOS than those without POPF. High-quality pancreaticojejunostomy performed by professional pancreatic surgeons and routine use of somatostatin after surgery are the key to reducing POPF and improving survival [60, 61]..

In 2007, Gockel [62] first expounded the concept of mesopancreas. In 2012, Adham and Singhirunnusorn [63] further proposed the concept of TMpE, and refined the anatomical part of the mesopancreas into the "mesopancreas triangle", and TMpE has gradually attracted attention in the field of pancreatic surgery, but at the anatomical and histopathological levels, the concept of "mesopancreas" is still very controversial. However many studies [63, 64] have confirmed that TMpE guided by the concept of "mesopancreas" can increase the resection rate of nerves, blood vessels, lymph nodes and other tissues behind the pancreatic head, thereby increasing the R0 resection rate of PHC. TMpE's operation time, intraoperative blood loss, incidence of postoperative complications, hospital stay, and perioperative mortality are all comparable to other current surgical methods for PHC, indicating that the surgical safety of TMpE is reliable in large pancreatic centers [63]. Our results showed that there was no statistical difference in mOS between TMpE and PD, indicating that TMpE cannot significantly improve the prognosis of PHC. In view of the special biological behavior of PC, whether the application of TMpE technology can improve the survival requires further study [65].

Some studies confirmed the effect of postoperative adjuvant chemotherapy and different regimens on the prognosis of PC [66, 67]. If there is no contraindication, patients with better postoperative physical recovery should receive adjuvant chemotherapy within 8 weeks postoperatively, reaching six courses or more [68, 69]. Our study showed that postoperative adjuvant chemotherapy is an independent prognostic factor in the PC, PHC, and PBTC subgroups. Only 56 patients (9.0%) received postoperative adjuvant chemotherapy in our study, which was significantly lower than those reported in relevant literatures, reflecting the insufficient emphasis on postoperative adjuvant chemotherapy.

## Conclusions

Therefore, PC has a poor prognosis and short survival. Early diagnosis and appropriate treatment are essential for prognosis. According to the preoperative and postoperative clinicopathological features, through MDT, a precise and optimized comprehensive treatment scheme is formulated to improve the quality of life and prolong the patient’s survival.

Limitations of this study: (1) The number of cases diagnosed by ultrasound-guided needle biopsy is small, and the molecular morphology information is less, and it is impossible to further study its influence on the prognosis. In the future, it is necessary to strengthen the acquisition of molecular morphological information of puncture pathology in order to further study its influence on prognosis. (2) The number of lymph nodes retrieved from the resected specimens is small, so it is difficult to analyze the correlation between the number of lymph nodes retrieved and prognosis, which is a shortcoming in our work. we need to increase the ability to retrieve lymph nodes in future work. (3) Neoadjuvant chemotherapy was not performed for borderline resectable PC, and the number of patients who underwent postoperative adjuvant chemotherapy was small. In the future, attention should be paid to preoperative neoadjuvant chemotherapy and postoperative adjuvant chemotherapy to prolong survival as much as possible.

## Data Availability

The data that support the findings of this study are available on request from the corresponding author. The data are not publicly available due to privacy or ethical restrictions.

## References

[1] Chen W (2015). Cancer statistics: updated cancer burden in China. Chin J Cancer Res.

[2] Rahib L, Smith BD, Aizenberg R, Rosenzweig AB, Fleshman JM, Matrisian LM (2014). Projecting cancer incidence and deaths to 2030: the unexpected burden of thyroid, liver, and pancreas cancers in the United States. Cancer Res.

[3] Sirri E, Castro FA, Kieschke J, Jansen L, Emrich K, Gondos A (2016). Recent trends in survival of patients with pancreatic cancer in germany and the United States. Pancreas.

[4] Worni M, Guller U, White RR, Castleberry AW, Pietrobon R, Cerny T (2013). Modest improvement in overall survival for patients with metastatic pancreatic cancer: a trend analysis using the surveillance, epidemiology, and end results registry from 1988 to 2008. Pancreas.

[5] Winter JM, Cameron JL, Campbell KA, Arnold MA, Chang DC, Coleman J (2006). 1423 pancreaticoduodenectomies for pancreatic cancer: a single-institution experience. J Gastrointest Surg.

[6] Benassai G, Mastrorilli M, Quarto G, Cappiello A, Giani U, Mosella G (2000). Survival after pancreaticoduodenectomy for ductal adenocarcinoma of the head of the pancreas. Chir Ital.

[7] Balaban EP, Mangu PB, Khorana AA, Shah MA, Mukherjee S, Crane CH (2016). Locally advanced, unresectable pancreatic cancer: american society of clinical oncology clinical practice guideline. J Clin Oncol.

[8] Vaccaro V, Sperduti I, Vari S, Bria E, Melisi D, Garufi C (2015). Metastatic pancreatic cancer: Is there a light at the end of the tunnel?. World J Gastroenterol.

[9] Conroy T, Desseigne F, Ychou M, Bouche O, Guimbaud R, Becouarn Y (2011). FOLFIRINOX versus gemcitabine for metastatic pancreatic cancer. N Engl J Med.

[10] Von Hoff DD, Ervin T, Arena FP, Chiorean EG, Infante J, Moore M (2013). Increased survival in pancreatic cancer with nab-paclitaxel plus gemcitabine. N Engl J Med.

[11] Tempero MA, Malafa MP, Al-Hawary M, Asbun H, Bain A, Behrman SW (2017). Pancreatic adenocarcinoma, version 2.2017, NCCN clinical practice guidelines in oncology. J Natl Compr Canc Netw.

[12] Bassi C, Marchegiani G, Dervenis C, Sarr M, Abu Hilal M, Adham M (2017). The 2016 update of the International Study Group (ISGPS) definition and grading of postoperative pancreatic fistula: 11 Years After. Surgery.

[13] Lim JE, Chien MW, Earle CC (2003). Prognostic factors following curative resection for pancreatic adenocarcinoma: a population-based, linked database analysis of 396 patients. Ann Surg.

[14] DiMagno EP, Reber HA, Tempero MA (1999). AGA technical review on the epidemiology, diagnosis, and treatment of pancreatic ductal adenocarcinoma. American Gastroenterological Association. Gastroenterology.

[15] Raghavan SR, Ballehaninna UK, Chamberlain RS (2013). The impact of perioperative blood glucose levels on pancreatic cancer prognosis and surgical outcomes: an evidence-based review. Pancreas.

[16] Cheon YK, Koo JK, Lee YS, Lee TY, Shim CS (2014). Elevated hemoglobin A1c levels are associated with worse survival in advanced pancreatic cancer patients with diabetes. Gut Liver.

[17] Dalal S, Hui D, Bidaut L, Lem K, Del Fabbro E, Crane C (2012). Relationships among body mass index, longitudinal body composition alterations, and survival in patients with locally advanced pancreatic cancer receiving chemoradiation: a pilot study. J Pain Symptom Manage.

[18] Bachmann J, Heiligensetzer M, Krakowski-Roosen H, Buchler MW, Friess H, Martignoni ME (2008). Cachexia worsens prognosis in patients with resectable pancreatic cancer. J Gastrointest Surg.

[19] Siddiqui A, Heinzerling J, Livingston EH, Huerta S (2007). Predictors of early mortality in veteran patients with pancreatic cancer. Am J Surg.

[20] Sakamoto T, Yagyu T, Uchinaka E, Miyatani K, Hanaki T, Kihara K (2020). Sarcopenia as a prognostic factor in patients with recurrent pancreatic cancer: a retrospective study. World J Surg Oncol.

[21] Yamada S, Shimada M, Morine Y, Imura S, Ikemoto T, Saito Y (2021). Significance of frailty in prognosis after surgery in patients with pancreatic ductal adenocarcinoma. World J Surg Oncol.

[22] Roxburgh CS, McMillan DC (2010). Role of systemic inflammatory response in predicting survival in patients with primary operable cancer. Future Oncol.

[23] Lin S, Fang Y, Mo Z, Lin Y, Ji C, Jian Z (2020). Prognostic value of lymphocyte to monocyte ratio in pancreatic cancer: a systematic review and meta-analysis including 3338 patients. World J Surg Oncol.

[24] Sierzega M, Lenart M, Rutkowska M, Surman M, Mytar B, Matyja A (2017). Preoperative neutrophil-lymphocyte and lymphocyte-monocyte ratios reflect immune cell population rearrangement in resectable pancreatic cancer. Ann Surg Oncol.

[25] Mowbray NG, Griffith D, Hammoda M, Shingler G, Kambal A, Al-Sarireh B (2018). A meta-analysis of the utility of the neutrophil-to-lymphocyte ratio in predicting survival after pancreatic cancer resection. HPB (Oxford).

[26] Takamori H, Hiraoka T, Kanemitsu K, Tsuji T, Hamada C, Baba H (2006). Identification of prognostic factors associated with early mortality after surgical resection for pancreatic cancer--under-analysis of cumulative survival curve. World J Surg.

[27] Lee SR, Kim HO, Son BH, Yoo CH, Shin JH (2013). Prognostic factors associated with long-term survival and recurrence in pancreatic adenocarcinoma. Hepatogastroenterology.

[28] Nakata B, Amano R, Kimura K, Hirakawa K (2013). Comparison of prognosis between patients of pancreatic head cancer with and without obstructive jaundice at diagnosis. Int J Surg.

[29] Tomasello G, Ghidini M, Costanzo A, Ghidini A, Russo A, Barni S (2019). Outcome of head compared to body and tail pancreatic cancer: a systematic review and meta-analysis of 93 studies. J Gastrointest Oncol.

[30] Lai IR, Lee WJ, Huang MT, Lin HH (2002). Comparison of serum CA72-4, CEA, TPA, CA19-9 and CA125 levels in gastric cancer patients and correlation with recurrence. Hepatogastroenterology.

[31] Hartwig W, Strobel O, Hinz U, Fritz S, Hackert T, Roth C (2013). CA19-9 in potentially resectable pancreatic cancer: perspective to adjust surgical and perioperative therapy. Ann Surg Oncol.

[32] Chen Y, Gao SG, Chen JM, Wang GP, Wang ZF, Zhou B (2015). Serum CA242, CA199, CA125, CEA, and TSGF are Biomarkers for the Efficacy and Prognosis of Cryoablation in Pancreatic Cancer Patients. Cell Biochem Biophys.

[33] Lee KJ, Yi SW, Chung MJ, Park SW, Song SY, Chung JB (2013). Serum CA 19-9 and CEA levels as a prognostic factor in pancreatic adenocarcinoma. Yonsei Med J.

[34] Modolell I, Guarner L, Malagelada JR (1999). Vagaries of clinical presentation of pancreatic and biliary tract cancer. Ann Oncol.

[35] Artinyan A, Soriano PA, Prendergast C, Low T, Ellenhorn JD, Kim J (2008). The anatomic location of pancreatic cancer is a prognostic factor for survival. HPB (Oxford).

[36] Watanabe I, Sasaki S, Konishi M, Nakagohri T, Inoue K, Oda T (2004). Onset symptoms and tumor locations as prognostic factors of pancreatic cancer. Pancreas.

[37] Lau MK, Davila JA, Shaib YH (2010). Incidence and survival of pancreatic head and body and tail cancers: a population-based study in the United States. Pancreas.

[38] Shoup M, Conlon KC, Klimstra D, Brennan MF (2003). Is extended resection for adenocarcinoma of the body or tail of the pancreas justified?. J Gastrointest Surg.

[39] Christein JD, Kendrick ML, Iqbal CW, Nagorney DM, Farnell MB (2005). Distal pancreatectomy for resectable adenocarcinoma of the body and tail of the pancreas. J Gastrointest Surg.

[40] van Geenen RC, Keyzer-Dekker CM, van Tienhoven G, Obertop H, Gouma DJ (2002). Pain management of patients with unresectable peripancreatic carcinoma. World J Surg.

[41] McKay A, Mackenzie S, Sutherland FR, Bathe OF, Doig C, Dort J (2006). Meta-analysis of pancreaticojejunostomy versus pancreaticogastrostomy reconstruction after pancreaticoduodenectomy. Br J Surg.

[42] Wright GP, Poruk KE, Zenati MS, Steve J, Bahary N, Hogg ME (2016). Primary tumor resection following favorable response to systemic chemotherapy in stage iv pancreatic adenocarcinoma with synchronous metastases: a bi-institutional analysis. J Gastrointest Surg.

[43] Tachezy M, Gebauer F, Janot M, Uhl W, Zerbi A, Montorsi M (2016). Synchronous resections of hepatic oligometastatic pancreatic cancer: Disputing a principle in a time of safe pancreatic operations in a retrospective multicenter analysis. Surgery.

[44] Shrikhande SV, Kleeff J, Reiser C, Weitz J, Hinz U, Esposito I (2007). Pancreatic resection for M1 pancreatic ductal adenocarcinoma. Ann Surg Oncol.

[45] Gleisner AL, Assumpcao L, Cameron JL, Wolfgang CL, Choti MA, Herman JM (2007). Is resection of periampullary or pancreatic adenocarcinoma with synchronous hepatic metastasis justified?. Cancer.

[46] Frigerio I, Regi P, Giardino A, Scopelliti F, Girelli R, Bassi C (2017). Downstaging in stage iv pancreatic cancer: a new population eligible for surgery?. Ann Surg Oncol.

[47] Yamada H, Hirano S, Tanaka E, Shichinohe T, Kondo S (2006). Surgical treatment of liver metastases from pancreatic cancer. HPB (Oxford).

[48] Ren Y, Dai C, Zheng H, Zhou F, She Y, Jiang G (2016). Prognostic effect of liver metastasis in lung cancer patients with distant metastasis. Oncotarget.

[49] Oweira H, Petrausch U, Helbling D, Schmidt J, Mehrabi A, Schob O (2017). Prognostic value of site-specific extra-hepatic disease in hepatocellular carcinoma: a SEER database analysis. Expert Rev Gastroenterol Hepatol.

[50] Dong F, Shen Y, Gao F, Xu T, Wang X, Zhang X (2017). Prognostic value of site-specific metastases and therapeutic roles of surgery for patients with metastatic bladder cancer: a population-based study. Cancer Management and Research.

[51] Wagner M, Redaelli C, Lietz M, Seiler CA, Friess H, Buchler MW (2004). Curative resection is the single most important factor determining outcome in patients with pancreatic adenocarcinoma. Br J Surg.

[52] Kulkarni NM, Soloff EV, Tolat PP, Sangster GP, Fleming JB, Brook OR, et al. White paper on pancreatic ductal adenocarcinoma from society of abdominal radiology's disease-focused panel for pancreatic ductal adenocarcinoma: Part I, AJCC staging system, NCCN guidelines, and borderline resectable disease. Abdom Radiol. 2020;45:716–28. 10.1007/s00261-019-02289-5 31748823

[53] Tang K, Lu W, Qin W, Wu Y (2016). Neoadjuvant therapy for patients with borderline resectable pancreatic cancer: a systematic review and meta-analysis of response and resection percentages. Pancreatology.

[54] Crino SF, Di Mitri R, Nguyen NQ, Tarantino I, de Nucci G, Deprez PH (2021). Endoscopic ultrasound-guided fine-needle biopsy with or without rapid on-site evaluation for diagnosis of solid pancreatic lesions: a randomized controlled non-inferiority trial. Gastroenterology.

[55] Xie IY, Gallinger S (2020). The genomic landscape of recurrent pancreatic cancer is modified by treatment. Nat Rev Gastroenterol Hepatol.

[56] Sakamoto H, Attiyeh MA, Gerold JM, Makohon-Moore AP, Hayashi A, Hong J (2020). The Evolutionary Origins of Recurrent Pancreatic Cancer. Cancer Discov.

[57] Nagai S, Fujii T, Kodera Y, Kanda M, Sahin TT, Kanzaki A (2011). Recurrence pattern and prognosis of pancreatic cancer after pancreatic fistula. Ann Surg Oncol.

[58] Vollmer CM, Sanchez N, Gondek S, McAuliffe J, Kent TS, Christein JD (2012). A root-cause analysis of mortality following major pancreatectomy. J Gastrointest Surg.

[59] Bassi C, Dervenis C, Butturini G, Fingerhut A, Yeo C, Izbicki J (2005). Postoperative pancreatic fistula: an international study group (ISGPF) definition. Surgery.

[60] Shrikhande SV, Sivasanker M, Vollmer CM, Friess H, Besselink MG, Fingerhut A (2017). Pancreatic anastomosis after pancreatoduodenectomy: A position statement by the International Study Group of Pancreatic Surgery (ISGPS). Surgery.

[61] Gurusamy KS, Koti R, Fusai G, Davidson BR. Somatostatin analogues for pancreatic surgery. Cochrane Database Syst Rev. 2013;4:CD008370. 10.1002/14651858.CD008370.pub3 PMC7175835 23633353

[62] Gockel I, Domeyer M, Wolloscheck T, Konerding MA, Junginger T (2007). Resection of the mesopancreas (RMP): a new surgical classification of a known anatomical space. World J Surg Oncol.

[63] Adham M, Singhirunnusorn J (2012). Surgical technique and results of total mesopancreas excision (TMpE) in pancreatic tumors. Eur J Surg Oncol.

[64] Inoue Y, Saiura A, Yoshioka R, Ono Y, Takahashi M, Arita J (2015). Pancreatoduodenectomy With Systematic Mesopancreas Dissection Using a Supracolic Anterior Artery-first Approach. Ann Surg.

[65] Chowdappa R, Challa VR (2015). Mesopancreas in pancreatic cancer: where do we stand - review of literature. Indian J Surg Oncol.

[66] Oettle H, Neuhaus P, Hochhaus A, Hartmann JT, Gellert K, Ridwelski K (2013). Adjuvant chemotherapy with gemcitabine and long-term outcomes among patients with resected pancreatic cancer: the CONKO-001 randomized trial. JAMA.

[67] Neoptolemos JP, Palmer DH, Ghaneh P, Psarelli EE, Valle JW, Halloran CM (2017). Comparison of adjuvant gemcitabine and capecitabine with gemcitabine monotherapy in patients with resected pancreatic cancer (ESPAC-4): a multicentre, open-label, randomised, phase 3 trial. Lancet.

[68] Khorana AA, Mangu PB, Berlin J, Engebretson A, Hong TS, Maitra A (2017). Potentially Curable Pancreatic Cancer: American Society of Clinical Oncology Clinical Practice Guideline Update. J Clin Oncol.

[69] Khorana AA, Mangu PB, Berlin J, Engebretson A, Hong TS, Maitra A (2016). Potentially Curable Pancreatic Cancer: American Society of Clinical Oncology Clinical Practice Guideline. J Clin Oncol.

